# A Tale of Two Solitudes: Loneliness and Anxiety of Family Caregivers Caring in Community Homes and Congregate Care

**DOI:** 10.3390/ijerph181910010

**Published:** 2021-09-23

**Authors:** Sharon Anderson, Jasneet Parmar, Bonnie Dobbs, Peter George J. Tian

**Affiliations:** 1 Division of Care of the Elderly, Department of Family Medicine, University of Alberta, Edmonton, AB T5G 2T4, Canada; Jasneet.parmar@albertahealthservices.ca (J.P.); bdobbs@ualberta.ca (B.D.); peter.tian@ualberta.ca (P.G.J.T.); 2 Home Living Edmonton Zone, Alberta Health Services, Edmonton, AB T5G OB7, Canada; 3 Medically At-Risk Driver Centre, University of Alberta Edmonton, Edmonton, AB T5G 2T4, Canada

**Keywords:** family caregivers, carers, loneliness, anxiety, COVID-19

## Abstract

We surveyed 604 family caregivers residing in the province of Alberta to better understand the impact of the COVID-19 pandemic on anxiety, loneliness, and care work. We assessed anxiety with the Six-Item State Anxiety Scale and loneliness with the DeJong-Gierveld Loneliness Scale. The COVID-19 pandemic created two contexts giving rise to feelings of solitude for family caregivers. Family caregivers of Albertans living in private community homes were overwhelmed with caregiving needs while those caring for Albertans living in congregate settings were restricted from caregiving. The results indicated that before the COVID-19 pandemic, 31.7% of family caregivers were anxious and 53.5% were lonely. The proportions of those who were anxious rose to 78.8% and lonely to 85.9% during the pandemic. The qualitative responses of family caregivers connected being overwhelmed with care work either in community homes or as the designated essential caregiver in congregate living settings, as well as being unable to care in congregate care settings, with anxiety and loneliness. The caregivers reporting improvements in their health and relationships with care-receivers credited spending time with the receiver doing pleasant activities together, rather than purely performing onerous care tasks. Policymakers need to consider organizing health and community services to ensure family caregivers are not overwhelmed with care tasks or excluded from caring in congregate care.

## 1. Introduction

In 2018, Statistics Canada reported 7.8 million Canadians aged 15 and older (25% of the population) were family caregivers [1]. We define family caregiver (carer, care-partner) broadly as any person who takes on a generally unpaid caring role providing emotional, physical, or practical support in response to an illness, disability, or age-related need. Family caregivers care in hospitals, their homes, the care receivers’ residence, and in congregate care settings such as group homes, assisted or supportive living, and long-term care. Family caregivers have been critical to keeping care receivers who were at particularly high-risk of severe infection and mortality from COVID-19 safe and supported emotionally [2,3]. However, the impact on family caregivers and their related needs have been largely ignored in pandemic responses to date [4].

Although family caregiving can be onerous at the best of times, it has become more challenging because of the COVID-19 pandemic [5,6]. Carers UK, for example, reported that a month after the pandemic was declared, 70% of the 5047 family caregivers completing their survey were providing more care than before the COVID-19 pandemic public health protocols were initiated [6].

Prior to the-COVID-19 pandemic, family caregivers provided 75–90% of the care to persons living with frailty, complex chronic conditions, and impairments in the community [7,8,9], and assisted with 10 to 30% of the care for congregate care residents [10,11]. After the World Health Organization declared that COVID-19 was a pandemic on 11 March 2020 [12], family caregivers caring for people residing in community homes reported that their work had escalated and was being complicated by premature patient discharges, restricted admissions to long-term care, and reducing home care services and isolating at home [5,6].

The situation was reversed for family caregivers of congregate care residents. At the outset of the COVID-19 pandemic, they were completely restricted from entering congregate care settings (assisted/supportive living, long-term care, hospitals, group homes). COVID-19 infections, however, still broke out in long-term care. In fact, long-term care residents were far more likely to be infected and die of COVID-19 than older people living in community homes [13,14]. However, the health of supportive living and long-term care residents deteriorated due to isolation.

As the COVID-19 pandemic continued, some jurisdictions have eased restrictions and allowed some family caregivers to assist with hands-on care, whereas others continued with restrictions [3]. Similarly, some in-person health and community services (e.g., respite, home support) resumed and others (support programs) were offered online. Therefore, we aimed to evaluate the impacts of the COVID-19 pandemic and the public health protocols to control COVID-19 on family caregivers’ work, loneliness, anxiety, and self-rated mental and physical health early in the pandemic. We surveyed caregivers again in July 2021 (one year after the initial survey). In what follows, we briefly review family caregiver work, anxiety, and loneliness prior to the COVID-19 pandemic then report on our survey research.

### Family Caregiving Work, Anxiety, and Distress

Typically, caregiving becomes more onerous, and its consequences more acute, as the care receiver’s illness, frailty, and impairments progress [7,8]. In the last two decades, however, care work became even more complex and longer lasting due to medical advances, increased longevity, shorter hospital stays, and the push for community care. At the same time, increased participation in the labor force by women and the rising proportion of smaller, more geographically dispersed families have reduced the numbers of available family caregivers. [15,16]. Given these changes, the proportion of stressed caregivers has been increasing. In 2016, 33.3% of caregivers to long-term home care clients in Canada were distressed [17,18], rising from 15.6% in 2010 [19].

The transition to long-term care occurs when the care receiver’s care needs exceed the family caregiver’s capacities [20]. While the notion is that congregate care employees provide all the care, many family caregivers augment that paid care [10,20,21]. Family caregivers continue to provide emotional, social, and practical support including mental stimulation, direct care (assistance with grooming, dressing, mealtimes), money management, and indirect care (monitoring care, managing care, advocating) [10,20,21].

It is important to note that it is not family caregiving itself that is distressing; 88% of family caregivers to older parents say caring is rewarding [22]. Rather than distress from caregiving per se, anxiety is associated with being overwhelmed with care work and worry [7,8,23]. Family caregivers experiencing the most distress live with the care recipient, provide 20+ hours/week of care, and/or care for a person with more severe impairments, dementia, depression, and/or responsive behaviors [23]. All of these factors have been exacerbated by COVID-19, as resources to support family caregivers and the people they care for have been cut, reduced, or overtaxed during the pandemic [5,6].

We expected that feelings such as anxiety and loneliness would increase during COVID-19. Anxiety is an emotion characterized by feelings of tension, worried thoughts, and physical changes like increased blood pressure. Typically, anxiety increases as care responsibilities and exhaustion rise [24,25,26,27,28,29]. Prolonged low-level anxiety reduces concentration and increases fatigue and psychological confusion [30]. Thus, ongoing anxiety is a risk factor for increased disability, reduced quality of life, cognitive impairment, and premature mortality [25,30,31,32,33,34,35,36]. Recent meta-analyses estimated the pooled anxiety prevalence (pre-COVID-19) at 32.1% for family caregivers of people living with dementia [37], while Health Quality Ontario [17] reported considerably higher rates of distress for those caring for people with moderate to severe cognitive impairments (54.5%), extensive assistance with or dependent in some activities of daily living (48.7%), and complex chronic health needs/instability (56.1%).

Loneliness is defined as the discrepancy between the person’s expectations of the quantity or quality of relationships with others and the actuality of those relationships [38]. Loneliness is a risk factor for premature mortality and chronic conditions (heart disease, diabetes, depression, dementia) [39,40,41,42]. Prior to COVID-19, family caregivers were at greater risk of loneliness and social isolation than their non-caregiving counterparts [39,40,41,42]. A survey of American family caregivers reported that 42% of family caregivers were lonely compared to 34% of their mid- and older-life non-caregiving counterparts [43]. In the United Kingdom, Victor and colleagues [44] found two-thirds of their large sample (n = 1283) of family caregivers of people living with mild to moderate dementia were lonely (43.7% moderately lonely, 17.7% severely lonely).

Our objectives in this mixed methods survey research with family caregivers were to (1) learn about changes in family caregivers care work, (2) find out how much the anxiety and loneliness experienced by family caregivers had changed since the COVID-19 pandemic, and (3) understand family caregivers’ perceptions of their situation in the COVID-19 pandemic.

## 2. Materials and Methods

Canada confirmed its first case of COVID-19 on January 27, 2020, and there were just over 100 confirmed cases when the World Health Organization declared COVID-19 a pandemic on 11 March, 2020. Our research team, community not-for-profit association partners, and family caregivers designed the survey. Ten family caregivers not associated with the survey design pretested a paper-based version. After receiving Health Ethics Research Board approval (Pro00097996), the open format survey was offered online on the secure REDCap [45] data collection platform between 21 June and 31 July, 2020. Information about the survey, a word document of the survey, an invitation to be sent to family caregivers, and an online link to the survey were emailed to not-for-profit associations, seniors centers, Family and Community Support Services Program representatives and health care providers. Information about the survey and the online link was posted on social media (Facebook, Twitter, Instagram, LinkedIn).

People were asked two questions to qualify for inclusion in the survey, “Do you look after someone (or help to look after someone) who has a disability, mental illness, drug or alcohol dependency, chronic condition, dementia, or terminal or serious illness, who needs care due to frailty from aging, and/or COVID-19 without payment?” and “Do you live in Alberta?” Participants were informed that they were providing implied consent by continuing with the survey.

### 2.1. Survey Assessments

The survey sections used in this study consisted of three main sections: (1) care work, (2) self-rated anxiety, loneliness, and health, and (3) demographics (caregiver/care receiver). The full survey can be found in the Supplementary Materials (Supplementary File S1, Survey in Word).

Care work. We assessed the number of hours devoted to care work pre-COVID-19 with the following options < 9 h, 10–20 h, 21–39 h, and 40+ h. Family caregivers were asked whether care work increased, remained stable, or decreased during COVID-19. Those doing more care work were asked to estimate how many more hours per week they were required to work using the following categories: <9 h, 10–20 h, 21+ h.

Anxiety was assessed with the six-item State Anxiety Scale [46]. It is a validated short form of the State Trait Anxiety Inventory [STAI]. The long and short forms are designed to measure the feelings of apprehension, tension, nervousness, and worry. Family caregivers were asked about how they felt retrospectively, “Think back to before the COVID-19 pandemic, January 1, 2020, I felt comfortable” or “I felt good.” Later in the survey, they were asked “Right now, I feel comfortable” or “Right now, I feel good.” Participants responded to each of the items on a four-choice Likert scale with options ranging from “not at all” to “very much”. Items 1, 3, and 6 which are positively worded (absence of anxiety are reversed scored). The final score was obtained by adding the scores for each item together and then multiplying the total score by 20/6. STAI scores range from 20–80, with higher scores indicating more severe symptoms.

The six-item versions have been found to be as reliable and valid as the original 20 item version [46,47,48]. Cronbach alphas range from 0.74 to 0.82 [46]. In this survey, Cronbach’s alpha pre-COVID-19 was 0.85 and post-COVID-19 was 0.89. To permit comparability with previous studies, we dichotomized the STAI scores using cut point scores of <40 to indicate no or minimal symptoms and ≥41 to indicate the presence of moderate or severe symptoms.

Loneliness was measured with the six-item DeJong-Gierveld Loneliness Scale [49] retrospectively before the COVID-19 pandemic (1 January 2020) and currently (time of survey July 2020) [46]. While it was designed for use with older people, it has also been tested with large survey samples of adults 18 and over. There are three response categories: “Yes”, “More or less”, and “No”. The mix of positive, negative, and neutral responses avoids automatic answers and socially desirable responses. On the negatively worded items, the neutral (More or less) and positive answers (Yes) are scored as a 1 and No as 0. The positive questions are reverse scored. Total scores range from 1 to 6, where a score of 1 indicates no loneliness and 6 severe loneliness [49]. The scale is reliable and valid with reported Cronbach alphas ranging from 0.64 [38] to 0.74 [49]. In this survey, Cronbach’s alpha pre-COVID-19 was 0.77 and during COVID-19 was 0.76. As with previous studies [50,51], for ease in reporting proportions, we dichotomized the scale into not lonely ≤ 2 and lonely 2–6.

Self-rated changes in mental and physical health were assessed with the questions “Since we have had the public health COVID-19 pandemic restrictions (began 17 March 2020), my physical health has” and “Since we have had the public health COVID-19 pandemic restrictions (began 17 March 2020), my physical health has” rated on a three-point scale (Has improved, remained about the same, has deteriorated).

### 2.2. Qualitative Data Collection

In the survey, we included open-ended questions such as “Is there anything that you want to tell us about how your caregiving situation has changed since the COVID-19 pandemic began in March 2020?” “If you would like to, tell us more about how changes to home care services affected you or the person you care for.” and “If you have been affected by the COVID-19 visitation policies in lodges, supportive living, long-term care, auxiliary hospitals can you tell us more about your experience?”

### 2.3. Statistical Analysis

We used **SPSS**^®^ 26.0 statistical software (IBM^®^ Chicago, IL, USA) to analyze the data. We generated descriptive statistics for all variables. Prevalence was determined through calculating the proportions of family caregivers experiencing the dichotomized loneliness and anxiety scores pre-and during COVID. The pre- and during-COVID loneliness and anxiety means were compared with the paired T tests. ANOVA and the Tukey’s-Kramer post-hoc analysis was used to establish differences in the levels of anxiety and loneliness by receiver’s residence. To control for family-wise error rate, a *p* < 0.008 (*p* = 0.05/6 = 0.008) was considered significant.

### 2.4. Qualitative Analysis

Qualitative data were analyzed thematically using the stages outlined by Braun and Clarke [52]. Thematic analysis is a flexible qualitative method used to explore the different perspectives held by research participants, highlight the similarities and divergences in their viewpoints, and then generate thematic insights [52]. We methodically followed Braun and Clarke’s [52] six stages of analysis (see Supplementary File S2 Table of Stages of Thematic Analysis). Two members of the research team independently read qualitative responses to become familiar with the data and generate first impressions of meaning (Stage one). They made notes of impressions on the word transcripts. They discussed the initial impressions, then imported the data into NVivo. In stage two, they worked separately to inductively generate initial open codes (n = 158). Then, in stage three, team members worked together to generate categories (n = 25). They identified patterns within the open codes and grouped codes with similar attributes and meanings and then refined the categories into preliminary themes (Stage four) At this stage, discussing how the knowledge might apply in clinical practice and teamwork was useful for refining the overarching themes (n = 3). SA and JP reread the transcripts to verify and name the final themes (Stage five). The report was generated (Stage six) and discussed at a final team meeting. In this paper, we place the recipient’s living situation side by side in a table to illustrate the similarity and differences in family caregiver’s responses. Direct quotes are used illustrate participant viewpoints.

## 3. Results

In total, our recruitment strategy led to 1225 link click throughs from 21 June to 31 July, 2020. Only surveys with half of the survey questions completed were included in the subsequent analysis. This led to the rejection of incomplete data from some participants; 504 did not complete any questions (participation rate 58.9%) and 117 completed less than half the questions (completion rate 80.6%). No cookies or IP addresses were checked to prevent multiple entries; however, we did check manually and exclude identical entries. Margin of error is not applicable in this study due to the online recruitment methodology.

### 3.1. Participant Characteristics and Caregiving Situations

We analyzed the 604 surveys in which caregivers had completed over half of the questions. Most family caregivers were female (80.8%) and cared for one person (72.5%). About a third (29.4%) were under 54 years of age, a third (34.9%) 55 to 64 and a third 65 years and older (32.6). Pre-COVID-19, two-thirds (66.1%) cared for 20 or less hours a week and a third (33.3%) for more than 21 h a week. During the COVID-19 pandemic, half (50%) were providing more care, 16.1% were providing the same amount and, 33.9% were providing less care. Almost a quarter (21.3%) stated that they performed 10 or less hours of care weekly, 15.2% were contributing 11 to 20 h more a week, and 18.4% 21 or more hours a week.

The majority of family caregivers were caring for people living in community homes (54.3%) (FCG and care receiver live in same home, care receiver lives separate home), about a third for people in congregate care (29.4%) (lodges, assisted/supportive living, group home, long-term care), and 15.6% were providing care in more than one location (e.g., caring for two people residing in different settings, or for one person whose residence changed (e.g., community home to hospital to congregate care)). Just over half of caregivers rated the receiver’s frailty, health condition, or impairment as mild/moderate (53.5%) and 46.5% as severe (Table 1 shows the characteristics of the caregivers and care receivers).

### 3.2. Changes in Family Caregivers Care Work: Two Solitudes

**Figure 1:**
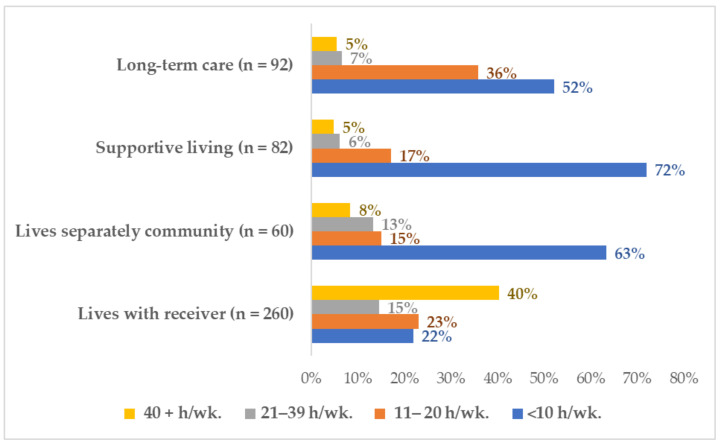
Pre-COVID-19 care work by receiver’s residence.

The COVID-19 pandemic has created two contexts giving rise to feelings of solitude for family caregivers. Solitude emphasizes the quality of being detached or separated from others. Family caregivers of Albertans living in private community homes were overwhelmed with caregiving needs, while those caring for Albertans living in congregate settings were restricted from caregiving. The family caregivers caring for someone who lived with them were providing the most care before the COVID-19 pandemic began (55.0% were providing 21 or more hours weekly), and they also performed the most care during the COVID-19 pandemic (see Figure 1). Almost a quarter (23.8%) provided 11 to 20 h a week of care, and over a third (36.3%) worked for 21 or more hours a week.

**Figure 2:**
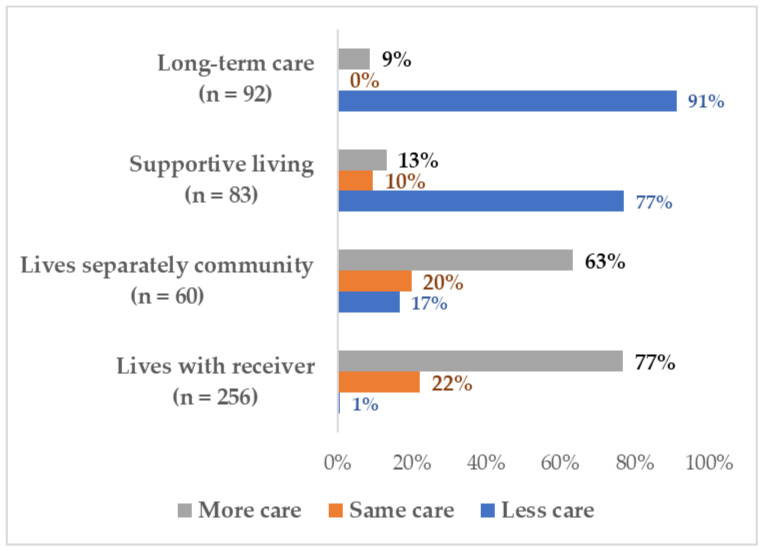
Proportion of caregivers providing more, equivalent, or less care by receiver residence during COVID-19.

At the other extreme, family caregivers of congregate living residents were prevented from entering these residences. Almost half (47.8%) of the family caregivers of long-term care residents had been caring for 11 or more hours a week before the COVID-19 pandemic, while the other half (52.2%) were providing 1 to 10 h a week of care. Almost all (91.3%) were unable to provide face-to-face care in the first four months of the COVID-19 pandemic (see Figure 2). Although 77.1% of the caregivers of supportive or assisted living residents were providing less face-to-face care, they were expected to continue to do the laundry, grocery shopping, and even monitor the resident’s medication taking without entering the facility. After 23 July 2020, the public health protocols in the province changed. Congregate care staff had discretion to allow a single person to be designated as an essential family caregiver to assist with resident care (e.g., personal care, meals, social support) (see Table 2 and Table 3 Care time by location).

The qualitative responses of family caregivers demonstrated that anxiety and loneliness have increased due to caregivers being overwhelmed with care work in community homes and being unable to care in congregate settings. In all care settings, family caregivers were worried about the care receiver’s wellbeing as social activities decreased. The family caregivers to people living in their own single-family home, apartment, or condo described diverse experiences. As schools, adult day programs, respite, and homecare supports closed, and family and friends social distanced; some family caregivers were caring for many more hours without any reprieve. Those caring for relatively independent receivers still living in their own home were likely to report that care stayed the same or they were caring for a few more hours doing tasks such as picking up groceries. However, some reported spending much more time together or increasing care hours. See qualitative quotes in Table 4.

Those reporting that care work had increased substantially described being anxious and isolated from others, whereas those providing similar levels of care or a few more hours a week wrote about feeling closer to the care-receiver (see Table 4). Moreover, the extra work combined with being isolated with the care receiver without interactions with other people increased relationship strain and anxiety. About a quarter of family caregivers reported that the extra costs of dealing with COVID-19 and lost income had left them in a financially precarious situation.

The family caregivers who were unable to provide care in supportive living or long-term care were particularly worried about the care receiver’s emotional and physical wellbeing. Over half commented that social isolation was exacerbating the resident’s cognitive and physical deterioration. They were distressed about missing precious time with care receivers who were at the end of life. See qualitative quotes in Table 4.

**Figure 3:**
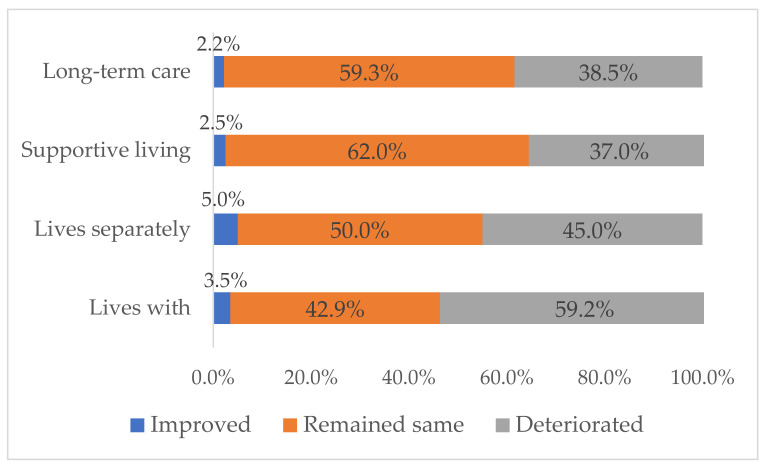
Caregiver’s Self-rated Changes in Physical Health.

Overall, just over half of the family caregivers (51.1%) thought their physical health had remained stable or improved, while 41.1% self-reported stable or improved mental health. The highest proportion of family caregivers reporting that their physical health had deteriorated were caring in their own home (see Figure 3). Some family caregivers in congregate care settings connected being overloaded with care work to the deterioration of their physical health. For example, one caregiver of a long-term care resident reported that her physical health had improved because the COVID-19 restrictions forced her to remain at home. The family caregivers allowed into congregate care settings as “essential” to the resident’s wellbeing reported that their physical health had deteriorated as hours of care increased. (See mental and physical health quotes in Table 4).

**Figure 4:**
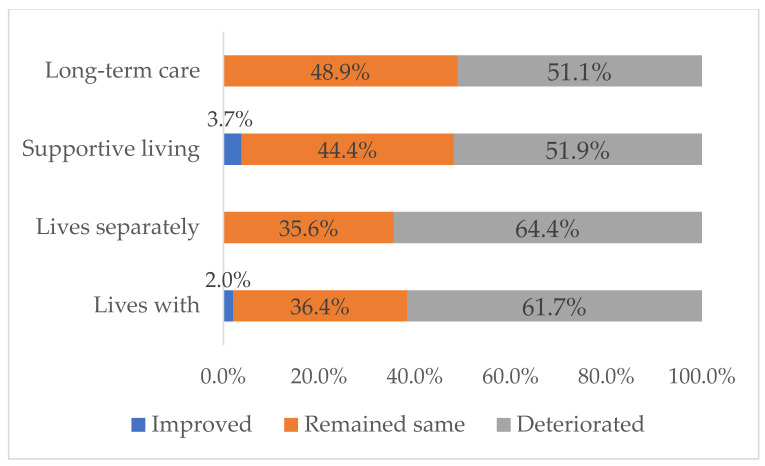
Caregiver’s Self-rated Changes in Mental Health.

A slightly higher proportion of the caregivers caring in community homes reported declines in mental health (Figure 4). They related deterioration in their mental health to extra care work and “being alone together” without face-to-face interactions with family, friends, and health and social care providers. Many reported that caregiver/receiver relationships had deteriorated from being alone together. In turn, relationship challenges increased the care-receiver’s and their own anxiety. Caregivers of supportive living and long-term care residents related declines in their mental health to worry about the care-receiver’s emotional and physical health.

### 3.3. Prevalence of Anxiety and Loneliness

**Figure 5:**
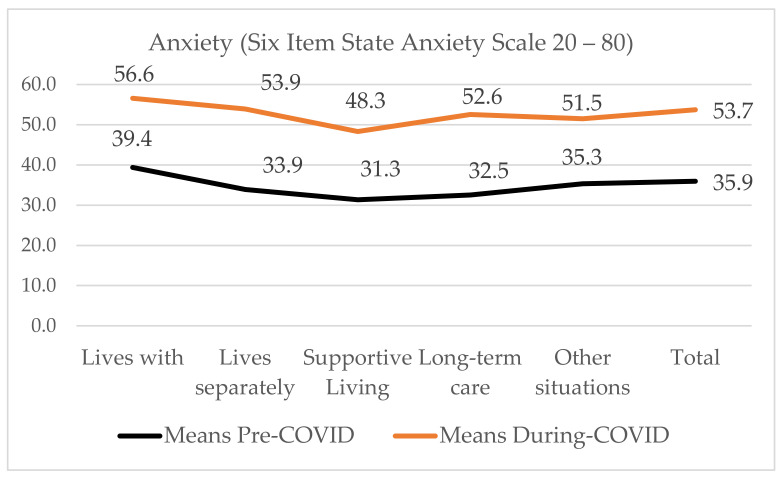
Mean anxiety by receiver’s residence.

Pre-COVID-19, 31.7% of family caregivers were anxious and 53.5% were lonely. During COVID-19, the proportion who were anxious rose to 78.8% and those who were lonely to 85.9% (see Table 5). On the one-way between-groups analysis to explore the impact of care receiver’s residence, we found a statistically significant difference in anxiety (*p* < 0.001) and loneliness (*p <* 0.001) (see Figure 5). Effect size calculated using eta squared was small, 0.04 for anxiety and 0.01 for loneliness. Post-hoc comparisons using the Tukey’s-Kramer test (Dunnet t, 2-sided) indicates anxiety was significantly different between those living with the receiver (M = 56.6, SD = 14.2), receivers who lived in supportive living (M = 48.3, SD = 13.5) while similar to those living in a separate community home (M = 53.9, SD = 15.3), in long-term care (M = 52.6, SD = 16.7) and other situations (M = 51.5, SD = 13.7).

**Figure 6:**
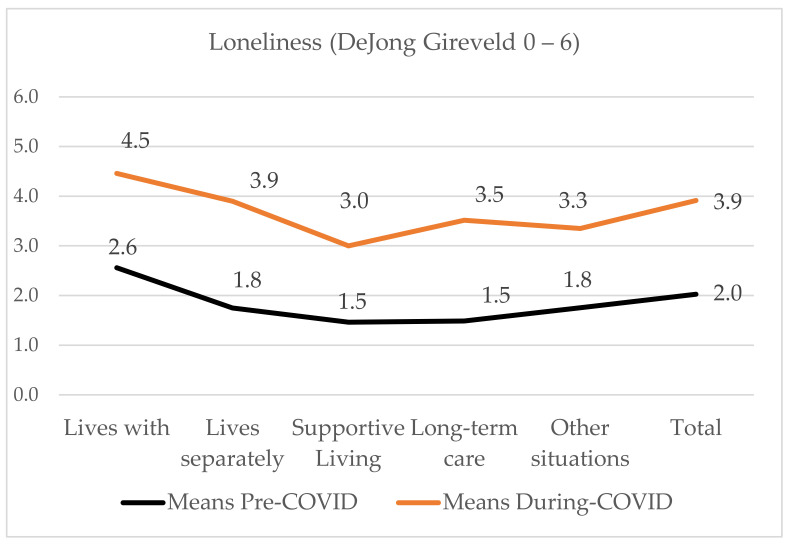
Mean loneliness by receivers’ residence.

Loneliness was similar for those in community homes either caring for a receiver who lived with them (m = 4.5, SD = 1.5) or living separately (m = 3.9, SD = 1.9). There were significant differences in loneliness for caregivers of receivers they lived with and those who lived in supportive living (m = 3.0, SD = 1.8), long-term care (m = 3.5, SD = 2.1) or in other situations (m = 3.3, SD = 1.8). See Figure 6.

### 3.4. Impact of COVID-19 on Anxiety and Loneliness

We used a paired t-test to evaluate the difference between the retrospectively measured pre-COVID-19 anxiety and loneliness ratings (January 1, 2020) and at the time of the survey (21 June–31 July, 2020). Table 4 shows statistically significant change in the Six-Item State Anxiety Scale [46] Pre (M = 35.77, SD = 12.42), Post (M = 53.57, SD 14.47), *p <* 0.0005, and the 6 Item DeJong-Gierveld Loneliness Scale [49]: Pre (M = 2.01, SD = 1.87), Post (M = 3.91, SD 1.85) *p* < 0.0005. The eta squared statistic indicated the effect size was moderate for anxiety (0.57) and for loneliness (0.50).

In the qualitative comments, caregivers described how anxiety and loneliness increased during the COVID-19 pandemic. Family caregivers caring at home and a few caring for someone in another community home used words like “overwhelmed”, “drowning”, or “exhausted” to describe the stress. Older caregivers wrote about how health problems of their own increased their anxiety, while working caregivers wrote about the stress of managing care work while supervising children’s virtual education and trying to manage working virtually from home.

Caregivers of congregate care residents were worried about residents being alone in their rooms and suffering from boredom. Window, phone, or virtual visits on ZOOM or Facetime worked better for supportive living residents than for frailer and typically more cognitively-impaired, long-term care residents. Dissatisfying window visits or virtual interactions increased family caregiver’s stress and guilt. Social distancing reduced emotional and practical support from family and friends which also exacerbated family caregiver’s loneliness. See Table 4 for exemplar quotes.

## 4. Discussion

The goal of this study was to examine the impacts of the COVID-19 pandemic and the public health protocols to control the spread of COVID-19 on family caregiver’s work, and to provide appraisals of their anxiety and loneliness. Family caregivers caring in community homes were providing more care than they had before the COVID-19 pandemic, whereas those caring for congregate care residents were prevented from providing in-person care. Measured retrospectively before the pandemic began (1 January, 2020) and four months after the COVID-19 pandemic was declared (July 2020), both anxiety and loneliness increased significantly. Family caregivers living with the receiver had the highest anxiety and loneliness means. Those caring for supportive living residents were the least anxious and lonely during the COVID-19 pandemic.

Long before the stress of the COVID-19 pandemic, population health researchers [30,40] and family caregiving scholars [53] were advising that prolonged anxiety, physically demanding caregiving, and ongoing loneliness could compromise caregiver’s physiological functioning and increase their risk for health problems [53,54,55]. We expected that both being overwhelmed with care in community homes without a break and worrying about a frail congregate care resident, many of whom were approaching the end of life, would increase anxiety. In their qualitative comments, family caregivers commented on how the extra care work and constant worry affected their health, particularly their mental health.

Anxiety is a common emotional response to worried feelings and tension [56]. It often manifests with poor concentration, insomnia, and elevated blood pressure [57], and is related to care burden [23,58]. In this study, family caregivers’ anxiety increased just four months into the COVID-19 pandemic. Family caregivers’ related anxiety to being unable to care and worrying about care-receivers’ wellbeing, as well as significant increases in weekly care hours along with deteriorating relationships or reduced social interactions. Data from the Canadian Resident Assessment Instrument-Homecare indicates that caregiver anxiety begins to rise dramatically once weekly care hours increase beyond 20 h per week [23,59]. Prolonged anxiety is associated with increased disability, reduced quality of life, cognitive impairment, and premature mortality [25,30,31,32,33,34,35,36].

Family caregivers also related loneliness to their health. We expected that the social and physical distancing intended to prevent the spread of COVID-19 could reduce the quantity and quality of social relationships [60,61]. While people like to spend some time alone or even prefer some isolation, the defining feature of loneliness is not enjoying being by yourself or the company of current companions [40,44]. The caregivers caring for community residents either in their own home or for receivers living in their own homes noted they were “on their own” as community and healthcare programs closed to reduce the risk of COVID-19 and reduce the strain of COVID-19 on the health system. Without interactions with others, caregivers felt that relationships with the care-receiver had deteriorated. Those caring for congregate living residents were unable to fulfill their caregiving role. Both groups felt excluded.

Even without the social distancing expected during COVID-19, moving into the caregiver role is associated with increases in loneliness [44,62]. In fact, in 2015, Carers UK found eight in 10 carers were lonely [63]. Herklots [64] noted that caregivers risk of loneliness increases as caregiving changes the relationship and caregivers disengage from other roles and social networks as hours of care increase. The caregiver’s role becomes limited to providing care, and relationships with health care providers become transactional as health providers focus on sustaining care rather than affirming family caregiver’s other roles [64]. The social relationships and respite that might have alleviated some of the family caregiver’s loneliness and anxiety were often unavailable during the COVID-19 pandemic.

### 4.1. Strengths and Limitations

Measuring anxiety and loneliness on validated scales is a strength of this study. Although a formal diagnosis of anxiety is achieved in a clinical interview, gathering data from self-reports is relatively common in studies of caregivers, as well as of people with chronic conditions. In fact, the State Anxiety Scale is one of the tools recommended for screening for caregiver anxiety by health care providers in community settings [46]. The assumption is that detecting anxiety and intervening should promote better quality of life.

We measured anxiety and loneliness retrospectively on 1 January 2020, and compared them to the caregivers’ perspectives regarding their current situations. Retrospective measurement is an important tool in the study of patient perceptions before an illness, but selection and recall biases are limitations [65]. While retrospective measurement may not be ideal, our pre-COVID-19 anxiety [37,66] and loneliness [43,44,63] measurements were similar to those reported in the academic literature. In 2016, Health Quality Ontario reported that, on average, 33.3% of caregivers were distressed [17], while the Senior’s Advocate in British Columbia found that figure to be 31% in 2017 [18]. Anderson and Thayer [43] reported that the prevalence of loneliness was 42% in an American population-based sample of caregivers, while Victor [44] found 61.4% of the UK caregivers participating in the Dementia and Enhancing Active Life study (2014–2016) were lonely. Victor also used the DeJong-Gierveld Loneliness Scale.

The majority (80.5%) of the participants were women. While one of the most consistent findings was that the majority of caregivers are female [67], recent studies are reporting that the proportion of male caregivers is increasing [1,15]. In Canada, men are more likely to take on financial, transportation, and home maintenance tasks whereas women are more likely to perform personal care and medical management which tend to be more intensive [53,68]. Women tend experience greater burden and impacts on psychological and physical health [53,68,69]. Thus, gender may partially explain the high proportion of caregivers indicating impacts on mental health in this study.

A strength of this study was tracing positive changes in caregivers’ mental and physical health to spending more time with care receivers in pleasant activities like reminiscing or learning the family history and less time in onerous care tasks. Caregiving scholars [70,71,72] advise exploring both the positive and negative aspects of caregiving. We structured our July 2021 survey to explore the positive aspects of caregiving on loneliness and anxiety in more depth.

The size of the survey sample may be a limitation. While a survey sample of over 600 appears large, online survey sample sizes tend to be larger. This survey was online for only 39 days during the summer when people spend more time outdoors than indoors at the computer. Our goal with this short timeline was to get a snapshot of family caregivers’ situations when the initial stringent COVID-19 protocols were put in place.

### 4.2. Implications

Publicity in the press and social media about the number of deaths and deterioration in the quality of care in long-term care during the COVID-19 pandemic shone a light on the essential family caregiver role in long-term care [3,4]. Notably, it clarified that family caregiving does not stop when receivers are admitted to congregate care, as well as emphasizing the benefits of family caregiver’s emotional, social, and practical support to residents. This study also illuminated just how much work family caregivers caring for people living in the community were doing before the pandemic, and the increase in care work due to the COVID-19 pandemic. Two in five (38.4%) of those caring for a receiver living with them were caring for more than 21 h per week, while 36% worked 21 or more hours a week after the pandemic began. Notably, anxiety has been shown to increase dramatically for family caregivers caring over 21 h a week. In future pandemics or flu outbreaks, health and community systems need to consider how to continue supportive services such as respite and personal care services that give family caregivers a break from caregiving, as well as to allow family caregivers to continue to care safely in congregate care settings.

This study can be regarded as in initial step to exploring the prevalence of loneliness and anxiety during the COVID-19 pandemic. We repeated this study in July 2021, and so will be able to better understand the evolution of anxiety and loneliness. We are also conducting qualitative interviews to explore other factors that would provide a fuller understanding of the elements that might be associated with increases in anxiety and loneliness.

## 5. Conclusions

The data from this study demonstrate that loneliness and anxiety rose in the first four months of the COVID-19 pandemic. In all care settings and throughout the care trajectory, we need to pay attention to family caregiver loneliness and anxiety, during the COVID-19 pandemic and beyond. In this pandemic, the plight of family caregivers prevented from caring in long-term care has been more visible, whereas the impacts of the COVID-19 public health protocols on family caregivers caring in private homes has been hidden. Mobilizing the knowledge from this research will help raise awareness of the importance of the role of family caregivers.

## Figures and Tables

**Table 1:** Demographics, and key caregiving characteristics.

Characteristics	n (%)
Caregiver age	604
15–34 years	30 (5.0)
35–54 years	147 (24.4)
55–64 years	211 (34.9)
65–74 years	154 (25.5)
75+ years Prefer not to answer	43 (7.1) 19 (3.1)
Caregiver sex	
Male	84 (13.9)
Female	488 (80.8)
Other Prefer not to answer	2 (0.3) 30 (5.0)
Care hours before COVID-19	
<less 10 h per week	255 (42.6)
11–20 h per week	142 (23.5)
21–39 h per week 40+ h per week Prefer not to answer	68 (11.3) 133 (22.0) 6 (1.0)
Changes in care since COVID-19	
More care	299 (50)
Same amount of care	96 (16.1)
Less care	203 (33.9)
Prefer not to answer	6 (1.0)
Care hours since COVID-19	
Less care	203 (34.1)
Same Amount of care	96 (16.1)
10 or less more hours per week	127 (21.3)
11–20 more hours per week	59 (15.2)
21–39 more hours per week	31 (5.1)
40+ more hours per week	79 (13.3)
Prefer not to answer	9 (2.0)
Caregivers’ physical health during COVID-19	
Improved Remained stable	25 (4.1) 284 (47.0)
Deteriorated	283 (46.9)
Prefer not to answer	12 (2.0)
Caregivers’ mental health during COVID-19	
Improved Remained stable	11 (1.8) 237 (39.2)
Deteriorated	339 (56.1)
Prefer not to answer	17 (2.9)
Number of people cared for	4
1 person	367 (72.5)
2 people	102 (20.2)
3 or more people	37 (7.3)
Prefer not to answer	98 (16.2)
Care receiver’s age	
Birth to-34 years	68 (11.3)
35–54 years	24 (4.0)
55–64 years	30 (5.0)
65–74 years	80 (13.2)
75+ years	259 (42.9)
Prefer not to answer	143 (23.7)
Care receiver’s living situation	
Same home/Lives with caregiver	262 (43.4)
Separate home/Condo/Apartment	60 (9.9)
Supportive/Assisted Living/Lodges	84 (13.9)
Long-term care	92 (15.5)
Other (two or more settings)	94 (15.6)
Prefer not to answer	12 (2.0)
Severity of receiver’s health condition	
Severe	105 (17.4)
Mild/moderate	121 (20.0)
Prefer not to answer	378 (62.6)

**Table 2:** Care hours per week (h/wk.) by care receiver’s living situation before the COVID-19 pandemic.

	<10 h/wk.		11–20 h/wk.		21–39 h/wk.		40+ h/wk.		Total	
	n	(%)	n	(%)	n	(%)	n	(%)	n	(%)
Lives with receiver	57	9.7	60	10.2	38	6.5	105	19.9	260	44.3
Lives separately community	38	6.5	9	1.5	8	1.4	5	0.9	60	10.2
Supportive living	59	10.1	14	2.4	5	0.9	4	0.7	82	14.2
Long-term care	48	8.2	33	5.6	6	1.0	5	0.9	92	15.7
Other	49	8.3	23	3.9	10	1.7	11	1.9	93	15.8
Total	251	42.8	139	12.7	67	11.4	130	22.1	587	100

**Table 3:** Care hours per week (h/wk.) by care receiver’s living situation during the COVID-19 pandemic.

	Less Care during COVID-19.		Same Care pre/during COVID-19.		<10 More h/wk.		11–20 More h/wk.		21+ More h/wk.		Total	
	n	(%)	n	(%)	n	(%)	n	(%)	n	(%)	n	(%)
Lives with receiver	2	0.8	57	22.3	43	16.8	61	23.8	93	36.3	256	43.8
Lives separately community	10	16.7	12	20.0	16	26.7	15	25.0	7	11.7	60	10.3
Supportive living	64	77.1	8	9.6	10	12.0	1	1.2	0	0	83	14.2
Long-term care	84	91.3	0	0	4	4.3	3	3.3	1	1.1	92	15.8
Other	39	41.9	17	18.3	20	21.5	10	10.8	7	7.5	93	15.9
Total	199	34.1	94	16.1	93	15.9	90	15.4	108	18.5	584	100.0

**Table 4:** Participants qualitative quotes by receiver’s residence.

Living with	Living Separately	Supportive Living	Long-Term Care
**Changes in family caregivers care work**			
This is much for difficult. The help I had developed is no longer able to assist. The few activities I had set up for my spouse are no longer available. On top of this, I also have my son with disabilities at home 24/7, as well. It feels like a dark hole, especially as both are cognitively impaired. The workload, the constant oversight and especially the lack of stimulation for me is really difficult now. Was trying to look for work and having interviews, but due to increase of care needed, and COVID-19, I am unbale to find work, and not qualified for any government financial help which adds to the current problems.	I am doing all the grocery shopping, prescription pick-ups, errands for my parents who are both in their 80’s to limit their community contacts. I’m feeling tired from all the extra assistance they need, but I would rather do this than increase their risk of community contact of COVID-19. I do not feel overburdened or anything. That’s quite a privilege. Yeah, personally, I feel like. I’m doing what a good daughter is supposed to do, because I do have friends who their parents are in care in a different community and talk to them once or twice a year. I just feel that I’m doing good.	At first, we talked on the phone regularly. It appeared as if he was doing well. When we started video chatting, it was very apparent how much my husband had gone downhill. He was always phoning me asking for things thinking that someone had stolen his stuff when he just couldn’t find it. I was unable to go help him get organized. He is on the second floor so difficult to see. It broke my heart to see him this week in an outside visit to see how much he has and I haven’t been there to help him. I am having health issues of my own I have been physically exhausted since my visit.	I can say with all honesty that after my mother was admitted to the long-term care facility and I could not visit, I could not sleep for the first 2+ weeks. I eventually had to use a prescribed sleep aid and sought the help of a psychologist as I simply could not stop thinking about my mother alone in a facility where she knew no one and she could not communicate her needs. Prior to COVID-19, I believe others would have considered me well adjusted, strong, resilient etc... I am saying these things, so that you would understand that I am not usually an anxious or nervous person.
**Anxiety**			
It’s overwhelming especially since he has fallen into depression since the Day Program has closed for now. I feel like I am drowning. For the last 7 years, I have been alone in my care. I am exhausted and concerned.	Family and friends are less available Homecare is felt to not be an option as services have been ceased in a number of cases Ongoing personal anxiety and bouts of feeling low and overwhelmed.	She lives in a seniors’ lodge facility and it was too confusing for her with window visiting and when we were able to visit outside. It was also extremely hard on us (daughters) and affected our mental health.	I’ve had a few sleepless nights worrying about her and wondering if she feels abandoned and what brightens up her day. I visualize her sitting, restrained in her wheelchair, with none of the little pleasures we all take for granted to brighten up the day.
**Loneliness**			
Isolated, on my own, support only by phone. Care is 24/7. I am getting less sleep. Self-care is nonexistent.	Social isolation placed more demand on me to be more emotionally, and socially available to my mother without any reprieve. Help from family and friends limited due to age and myriad of medical conditions of my mother. I feel inadequate.	Although I had peace of mind the staff were doing an awesome job at looking after my husband, it was difficult not knowing where my husband was mentally. Did he think I just abandoned him? Would he understand what was happening? I think we both are lonely.	My husband is placed in long-term [care]. Before I spent at least 4 days a week visiting, reading, walking, watching movies, playing games, going with him to activities. I miss being with him and I miss helping the other people and seeing their families.
**Changes in family caregiver’s mental and physical health**			
I am getting desperate. I have no life, gave it up 20 years ago when dad got sick, now mother...I have no life of my own, I am tired mentally and physically. I’m definitely wondering if I’m losing my mind many days as the COVID-19 atmosphere and spending my days with a mate who has dementia. The lack of social connection has been detrimental for both of us. I do need respite but realize that is not possible now. Stress levels are high, and I’ve been short tempered with him. All family caregivers need someone to openly talk too, no judgment.	When you when you end up in a care role, as in my situation, the physical and mental health care toll on the caregiver is enormous. During COVID-19, I know I’ve aged 20 years and COVID-19 has a lot to say about that because of the extensive increase in the amount of things I’m expected to do. But again, you asked earlier “has anybody ever asked how you’re doing?” No, not even my siblings. Like, nobody cares. Caring for my mom is a privilege. Before the COVID-19 pandemic, she was very independent and now my sister and I do the grocery shopping and we share a meal once or twice a week. My relationship with my mother has improved.	No contact with mother and I am the only child. I was brought up by my mom only since I was 6 years old. Feel upset and isolated from mom. I feel helpless. Being an essential visitor, I find that staff leans on me more for her care. I am unable to look for work as I seem to be on call for the times they can’t get her to eat, take her meds, or calm her down. It has been extremely hard on my mental, physical, and financial health. My mom and I are closer than we have ever been because it is just her and I. I have learned so much about her family. The staff at the lodge have been very accommodating.	The facility staff has done a tremendous job in managing the pandemic, but I need to visit my wife as she is slowly fading away from me. It makes me so sad. The sadness affects my health too. When my husband was living at home in January, I provided care 24/7 all year long. Homecare allowed me 6 h a week of respite. When my husband was first placed in LTC on Feb 4th, I travelled an hour each way to see him 5 days a week. I was terribly burned out but wanted to ensure he felt safe. The COVID pandemic forced me to stay at home. Physically, I regained my strength, but it was mentally challenging not to be able to see my husband.

**Table 5:** Anxiety and loneliness retrospectively before and during COVID-19: Means and dichotomized proportions by receivers’ residence.

	Anxiety Pre-COVID-19			Anxiety during COVID-19			Loneliness Pre-COVID-19			Loneliness during COVID-19		
	Scale 20–80	Not Anxious	Anxious	Scale 20–80	Not Anxious	Anxious	Scale 0–6	Not Lonely	Lonely	Scale 0–6	Not Lonely	Lonely
	Mean	n (%)	n (%)	Mean	n (%)	n (%)	Mean	n (%)	n (%)	Mean	n (%)	n (%)
Lives with/Same home	39.4	140 (56.9)	106 (43.1)	56.6	39 (15.4)	215 (84.6)	2.6	83 (32.9)	169 (67.1)	4.5	17 (6.7)	238 (93.3)
Lives in separate home	33.9	44 (73.3)	16 (26.7)	53.9	15 (25.9)	43 (74.1)	1.75	33 (55.0)	27 (45.0)	3.9	9 (15.0)	51 (85.0)
Supportive living	31.3	71 (86.6)	11 (13.4)	48.3	26 (32.9)	53 (67.1)	1.46	48 (60.0)	32 (40.0)	3.29	13 (16.3)	67 (83.3)
Long-term care	32.5	69 (78.4)	19 (21.6)	52.6	24 (27.3)	64 (72.7)	1.49	57 (64.8)	31 (35.2)	3.51	19 (21.3)	70 (78.7)
Other	35.3	64 (69.6)	28 (30.4)	51.5	17 (18.7)	74 (81.3)	1.8	44 (48.9)	46 (51.1)	3.3	23 (25.0)	69 (75.0)
Total	35.9	388 (68.3)	180 (31.7)	53.7	121 (21.2)	449 (78.8)	2.03	265 (46.5)	305 (53.5)	3.9	81 (14.1)	495 (85.9)

## Data Availability

Data belong to the survey Impact of COVID-19 on Family Caregivers in Alberta and the data are owned by Jasneet Parmar the principal investigator. People interested in obtaining the data set can contact Sharon Anderson, research coordinator of the study. Email sdanders@ualberta.ca.

## Supplementary Materials

The following are available online at https://www.mdpi.com/article/10.3390/ijerph181910010/s1, Supplementary File S1: Survey questionnaire, Supplementary File S2: Table Stages of Thematic Analysis, Supplementary File S3: CHERRIES Checklist for Reporting Results of Internet E-Surveys.
