# Supplementary material for: A Tale of Two Solitudes: Loneliness and Anxiety of Family Caregivers Caring in Community Homes and Congregate Care
Source: Int J Environ Res Public Health. 2021 Sep 23;18(19):10010. doi: 10.3390/ijerph181910010 (PMC8508318; doi:10.3390/ijerph181910010)
Supplement: Supplementary file 1 [file ijerph-18-10010-s001.zip › ijerph-1353872-supplementary.pdf]

Supplementary Materials 1: Survey of the impacts of COVID-19

We are inviting you to participate in a survey about how COVID-19 has affected caregivers in ALBERTA. It will take about 20 minutes of your time.

Principal Investigator: Dr. Jasneet Parmar Phone: 780-901-6236 Jasneet.parmar@ahs.ca
Affiliations: Department of Family Medicine, Faculty of Medicine and Dentistry, University of Alberta, (Edmonton, Alberta)
Funding: Northern Alberta Academic Family Medicine Fund

The COVID-19 pandemic has had a profound effect on all people in Canada, including family caregivers in Alberta. We define family *caregiver (carer, care-partner)* as any person who takes on a generally unpaid caring role providing emotional, physical or practical support in response to another person's illness, disability or age-related needs.

Why are you being asked to take part in this survey? You are a family caregiver in Alberta.

Study Purpose. We hope to understand the impact of the COVID-19 pandemic and public health social distancing measures on you, your workload, how you have managed and adapted, and your needs going forward. By sharing your opinions and experiences, you will help Dr Jasneet Parmar and her research team at the University of Alberta plan better supports for family caregivers in this COVID-19 pandemic and beyond.

How many people will participate in the survey? According to Statistics Canada data, there are almost a million family caregivers in Alberta. However, we do not know how many Alberta family caregivers will participate in the survey given their busy lives, but we estimate it could range between 100 and 1000 people.

What will happen if I participate in this survey? We are inviting family caregivers from Alberta to complete this voluntary survey. The survey should take you approximately 15 to 20 minutes depending on how you respond to questions. Your responses will remain anonymous. No personal or identifying information is being collected and all data will be analyzed as a group (aggregated). At the end of the survey, you will be asked whether you would like to be contacted for a follow-up interview about the impact of COVID-19 on you in your caregiving role. You can participate in this survey regardless whether you choose to be part of the follow-up study. You will also have the option to join the draw and win one of two $50 gift certificates! Participation in the draw is optional.

What are the potential risks or discomforts? Some questions that ask about how the COVID-19 physical distancing has affected you or the person you care for may be personally uncomfortable. Please note that you do not have to answer any questions you do not wish to answer or make you feel uncomfortable.

What are the potential benefits? There are no direct benefits to survey participants. However, the results of the survey may influence services to better support family caregivers.

Do I have to participate? Completing the survey is your choice. You have the option to not answer questions if you do not want to and continue the survey. You can exit the survey at any time by closing the survey. Because we are not collecting personal identifying information, we cannot remove the answers once you have completed the survey.

Will my information be kept confidential? Your responses to this survey are completely confidential. It will not be possible to identify you in any report. All data will be kept confidential and stored securely. We are using a secure online platform called REDCAP. Researcher access to the survey Is password-protected and transmission is encrypted. Survey responses cannot be linked to your data.

How long will the information be kept? In accordance with the University of Alberta data retention policy, we will keep the data for five years after completion of the study (2026).
If you have questions or concerns about this study, please contact any of the investigators.

Questions and Concerns:
If you have any questions about this survey, or if you would like us to mail to you a paper copy of the study please contact Dr. Sharon Anderson at 780-953- 5541 or sdanders@ualberta.ca

This study has received approval by the University of Alberta Health Ethics Research Board. If you have questions regarding your rights as a study participant, please call the University of Alberta Research Ethics Office at 780-492-2615.

By continuing with the survey your consent to participate is implied.

Section 1

1. Do you look after someone (or help look after someone) who has a disability, mental illness, drug or alcohol dependency, chronic condition, dementia, terminal or serious illness, who needs care due to frailty and aging or COVID-19?
   ☐Yes ☐No ☐Prefer not to answer/don’t know

2. Do you live in Alberta?
   ☐Yes ☐No ☐Prefer not to answer/don’t know

If you answered No to either of the two questions about—Thank you very much, at this time we are only surveying Alberta Caregivers

If you answered YES to the two questions above, you are a caregiver from Alberta, please continue with the survey.

Section 2 We have a few questions about the amount of care you provided and your other work before the COVID-19 pandemic (January 1, 2020) and since the COVID-19 pandemic took hold (March 2020)

Think back to BEFORE the COVID 19 pandemic (January 1, 2020).

In an average week, how much time did you spend providing care?

☐Less than an hour a week
☐1 to 5 hours a week
☐ 6-9 hours a week
☐10 to 20 hours a week
☐21 to 39 hours a week
☐More than 40 hours a week
☐Prefer not to answer/don’t know

Has the amount of care you provide to a family member, friend, or neighbor changed since the COVID-19 pandemic began (March 2020)?

☐ Yes, I am providing more care since the COVID- 19 pandemic began.

☐I am providing the same amount of care as I did before the COVID-19 pandemic.

☐I am providing less care than I was before the COVID-19 pandemic

☐Prefer not to answer/don't know

If you answered yes to question 2, you are providing MORE CARE since the COVID-19 pandemic, how many MORE hours of care are you providing per week?

☐Less than an hour a week

☐1 to 5 hours a week

☐6 to 9 hours a week.

☐10 hours a week

☐11 to 20 hours a week

☐21 to 39 hours a week

☐More than 40 hours a week

☒Prefer not to answer/don't know

1. If you are providing LESS since the COVID-19 pandemic, can you tell us more about why caring is taking less time since the COVID-19 pandemic began (March 2020)?

____

____

____

____

2. Is there anything that you want to tell us about how your caregiving situation has changed since the COVID-19 pandemic began in March 2020? Things to think about: Is the care more difficult or complex? Do you have the help you need from health care providers? Have your family and friends been able to help as they did before COVID-19. How do you feel?

____

____

____

____

3. What was your employment situation before the COVID-19 January 1st, 2020)?

☐ I worked part time

☐ I worked full time

☐ I was looking for work but not employed yet

☐I had given up my paid job because of my caregiving responsibilities

☐I had been unable to work for pay because of illness or disability

☐I was retired from the paid labor force

☐Prefer not to answer/don't know

Other____

____

4. What is your current employment situation? (Now since the COVID-19 pandemic began).

☐I am working from home

☐I am an essential worker, so I still go to work as normal

☐I am still going to work as normal

☐I am unable to work because of the current physical distancing rules

☐I have given up work because of my caregiving responsibilities.

☐I have been unable to work because of illness or disability.

☐I lost my job (laid off, let go, quit)

☐I am retired.

☐Prefer not to answer/don't know

☐Other

____

____

Section 3 Please help us to understand how you felt BEFORE the COVID- 19 Pandemic.

Select the response that best reflects how you felt on January 1, 2020

1. I often felt rejected before the COVID-19 pandemic (January 1, 2020).

   ☐Yes ☐ More or less ☐No ☐Prefer not to answer

2. I experienced a general sense of emptiness before the COVID-19 pandemic (January 1, 2020).

   ☐Yes ☐ More or less ☐No ☐Prefer not to answer

3. I missed having people around me before the COVID-19 pandemic (January 1, 2020).

   ☐Yes ☐ More or less ☐No ☐Prefer not to answer

4. There were plenty of people I could rely on when I had problems before the COVID-19 pandemic (January 1, 2020).

   ☐Yes ☐ More or less ☐No ☐Prefer not to answer

5. There were many people I could trust completely before the COVID-19 pandemic (January 1, 2020).

   ☐Yes ☐ More or less ☐No ☐Prefer not to answer

6. There were enough people I felt close to before the COVID-19 pandemic (January 1, 2020).

   ☐Yes ☐ More or less ☐No ☐Prefer not to answer

Please select the response that best reflects how you felt on January 1, 2020

7. I was comfortable

☒Not at all ☐Somewhat ☐Moderately ☐Very much ☐Prefer not to answer

8. I was anguished

☐Not at all ☐Somewhat ☐Moderately ☐Very much ☐Prefer not to answer

9. I felt at ease

   ☐Not at all ☐Somewhat ☐Moderately ☐Very much ☐Prefer not to answer

10. I felt nervous

    ☐Not at all ☐Somewhat ☐Moderately ☐Very much ☐Prefer not to answer

11. I felt concerned

    ☐Not at all ☐Somewhat ☐Moderately ☐Very much ☐Prefer not to answer

12. I felt good on January 1, 2020

    ☐Not at all ☐Somewhat ☐Moderately ☐Very much ☐Prefer not to answer

Section 4: Please help us to understand how you feel right now during the COVID-19 pandemic.

1. I often feel rejected (Right now, in the COVID-19 pandemic).

   ☐Yes ☐ More or less ☐No ☐Prefer not to answer

2. I experience a general sense of emptiness (Right now, in the COVID-19 pandemic).

   ☐Yes ☐ More or less ☐No ☐Prefer not to answer

3. I miss having people around me (Right now, in the COVID-19 pandemic).

   ☐Yes ☐ More or less ☐No ☐Prefer not to answer

4. There are plenty of people I can rely on when I have problems (Right now, in the COVID-19 pandemic).

   ☐Yes ☐ More or less ☐No ☐Prefer not to answer

5. There are enough people I feel close to (Right now, in the COVID-19 pandemic).

   ☐Yes ☐ More or less ☐No ☐Prefer not to answer

6. There are many people I can trust completely (Right now, in the COVID-19 pandemic).

   ☐Yes ☐ More or less ☐No ☐Prefer not to answer

7. I am comfortable (Right now, in the COVID-19 pandemic).

   ☐Not at all ☐Somewhat ☐Moderately ☐Very much ☐Prefer not to answer

8. I am anguished (Right now, in the COVID-19 pandemic).

   ☐Not at all ☐Somewhat ☐Moderately ☐Very much ☐Prefer not to answer

9. I feel at ease (Right now, in the COVID-19 pandemic).

   ☐Not at all ☐Somewhat ☐Moderately ☐Very much ☐Prefer not to answer

10. I feel nervous (Right now, in the COVID-19 pandemic).

    ☐Not at all ☐Somewhat ☐Moderately ☐Very much ☐Prefer not to answer

11. I feel concerned (Right now, in the COVID-19 pandemic).

    ☐Not at all ☐Somewhat ☐Moderately ☐Very much ☐Prefer not to answer

12. I feel good (Right now, in the COVID-19 pandemic).
    ☐Not at all ☐Somewhat ☐Moderately ☐Very much ☐Prefer not to answer
13. Since we have had the public health COVID-19 pandemic restrictions (began March 17, 2020), my MENTAL health has:
    ☐Has improved
    ☐Remained about the same
    ☐Has deteriorated (e.g., less fit, more worried, or more pain)
    ☐Other
    ☐Prefer not to answer
14. Since we have had the public health COVID-19 pandemic restrictions (began March 17, 2020), my PHYSICAL health has:
    ☐Has improved
    ☐Remained about the same
    ☐Has deteriorated (e.g., less fit, more worried, or more pain)
    ☐Other
    ☐Prefer not to answer
15. Since the COVID-19 pandemic, I am not able to take a break from caregiving.
    ☐Strongly Disagree ☐ Disagree ☐ Agree ☐ Strongly Agree
    ☐Prefer not to answer
16. Since the COVID-19 pandemic, I can't stop thinking about all the things I have to do.
    ☒Strongly Disagree ☐ Disagree ☐ Agree ☐ Strongly Agree
    ☒Prefer not to answer
17. I feel more frustrated since the COVID-19 pandemic
    ☒Strongly Disagree ☐ Disagree ☐ Agree ☐ Strongly Agree
    ☐Prefer not to answer
18. I am not sleeping well since the COVID-19 pandemic.
    ☒Strongly Disagree ☐ Disagree ☐ Agree ☐ Strongly Agree
    ☐Prefer not to answer

Section 5: The following questions are about your experiences with the health care system-- family doctors, hospital, homecare, or long-term care services/

1. Did you avoid visiting your doctor because you were worried about COVID-19?
   ☐Yes ☐No ☐Prefer not to answer

2. Did you avoid taking the person you care for to a doctor because you were worried about COVID-19?
   ☐Yes ☐No ☐Prefer not to answer
3. BEFORE the COVID-19 pandemic, has ANY health care professional asked you about your needs as a family caregiver?

☐Yes ☐No ☐Prefer not to answer

4. Before the COVID-19 pandemic began, who were the healthcare professionals who asked you about your caregiving situation or how you were doing as a caregiver?

   ☐My family doctor

   ☐The family doctor of the person I care for

   ☐A health professional in a hospital

   ☐A home care case manager

   Other (by profession) ______________________________

5. Since the COVID-19 pandemic began, has any health care professional asked you about your needs as a family caregiver?

   ☐Yes ☐No ☐Prefer not to answer

6. Since the COVID-19 pandemic began, who were the healthcare professionals who asked you about your caregiving situation or how you were as a caregiver?

   ☐My family doctor

   ☐The family doctor of the person I care for

   ☐A health professional in a hospital

   ☐A home care case manager

   Other (by profession) ______________________________

7. Do you currently have home care services for you or the person you care for?

   ☐Yes ☐No ☐Prefer not to answer

8. SINCE the COVID-19 pandemic began, has the home care case manager checked in with you or the person you care for?

   ☐Yes ☐No ☐Prefer not to answer

9. Have you or the person you cared for experienced any of the following changes with HOME CARE services since the COVID- 19 pandemic?

   ☐Services are the SAME as before COVID-19.

   ☐Receiving MORE services because the person I care for has higher needs

   ☐LESS service because of the COVID-19 pandemic

   ☐LESS service because the person I care for has fewer needs

   ☐I stopped services because I was worried about the person I care for getting COVID-19

   ☐Prefer not to answer

10. If you would like to, tell us more about how changes to home care services affected you or the person you care for.

____

____

____

____

Section 6 Now we want to ask you a few questions about the person or people that you care for.

1. How many people do you care for?

   ☐One person

   ☐Two people

   ☐Three people

   ☐Four or more people

   ☐Prefer not to answer

2. How old is the first person you care for?________________

3. How old is the second person you care for?______________

4. Where does the first person(s) you care for live? Check all that apply.

   ☐They live with you in the same household as you do.

   ☐They live separately in their own private household (house, condo, apartment)

   ☐They live with another family member or friend

   ☐They live in supportive living (e.g., lodge, assisted living)

   ☐They live in a long-term care home

   ☐They are in hospital

   ☐Other____

   ☐Prefer not to answer/don't know

What conditions, disabilities, or illnesses does the first person you care for have? Tick off all that apply.

   ☐Autism Spectrum Disorder

   ☐ Frailty due to aging

   ☐Acquired Brain Injury, Stroke, Head injury

   ☐Cancer

☐Chronic health condition (e.g., Diabetes, arthritis, asthma, Crohn's, Cystic Fibrosis, COPD)
☐COVID-19
☐Dementia
☐Drug or Alcohol dependency
☐Heart Disease
☐Intellectual disability
☐Mental Illness
☐Neurological condition (e.g., epilepsy, Parkinson's Disease, Multiple Sclerosis
☐Sensory impairment (e.g., hearing loss, vision loss, blindness)

☐Terminal or serious illness

Other Please add ____

____

5. Would you say the illness, impairment, frailty, health condition or COVID-19 of the first person you care for is mild, moderate or severe?
☐mild ☐moderate ☐severe ☐Prefer not to answer
6. How long can the first person you care for be left alone?
☐Not at all
☐Less than an hour
☐A few hours
☐One day
☐A few days
☐More than a few days.
☐Prefer not to answer
7. During the COVID-19 pandemic so far, the health of the first person I care for has:
☐Has improved

☐Remained about the same

☐Has deteriorated (e.g., more depressed, worried, forgetful, or frail)

☐Other____

☐Prefer not to answer

8. Where does the second person(s) you care for live? Check all that apply.
☐They live with you in the same household as you do.

☐They live separately in their own private household (house, condo, apartment)

☐They live with another family member or friend

☐They live in supportive living (e.g., lodge, assisted living)

☐They live in a long-term care home

☐They are in hospital

☐Other____

☐Prefer not to answer/don't know

9. What conditions, disabilities, or illnesses does the second person you care for have? Tick off all that apply

   ☐Autism Spectrum Disorder

   Frailty due to aging

   ☐Acquired Brain Injury, Stroke, Head injury

   ☐Cancer

   ☐Chronic health condition (e.g., Diabetes, arthritis, asthma, Crohn's, Cystic Fibrosis, COPD)

   ☐COVID-19

   ☐Dementia

   ☐Drug or Alcohol dependency

   ☐Heart Disease

   ☐Intellectual disability

   ☐Mental Illness

   ☐Neurological condition (e.g., epilepsy, Parkinson's Disease, Multiple Sclerosis

   ☐Sensory impairment (e.g., hearing loss, vision loss, blind)

   ☐Terminal or serious illness

   Other Please add ____

   ____

10. Would you say the illness, impairment, frailty, health condition or COVID-19 of the second person you care for is mild, moderate, or severe?

    ☐mild ☐moderate ☐severe ☐Prefer not to answer

11. How long can the second person you care for be left alone?

    ☐Not at all

    ☐Less than an hour

    ☐A few hours

    ☐One day

    ☐A few days

    ☐More than a few days.

    ☐Prefer not to answer

12. During the COVID-19 pandemic so far, the health of the second person I care for has

    ☐Has improved

    ☐Remained about the same

    ☐Has deteriorated (e.g., more depressed, worried, forgetful, or frail)

    ☐Other____

☐Prefer not to answer

13. Is there anything else that you want to tell us about the health of the person(s) you care for since the COVID-19 pandemic began (March 2020)?

____

____

____

____

14. If the person you care for lives in a lodge, supportive living, long-term care, or an auxiliary hospital, did you consider bringing the person you care for home because of the COVID- 19 pandemic?

☐Yes ☐No ☐Not applicable

15. If you have been affected by the COVID-19 visitation policies in lodges, supportive living, long-term care, auxiliary hospitals can you tell us more about your experience?

____

____

____

____

____

____

____

Section 7: Almost done! 3 Demographic Questions

400

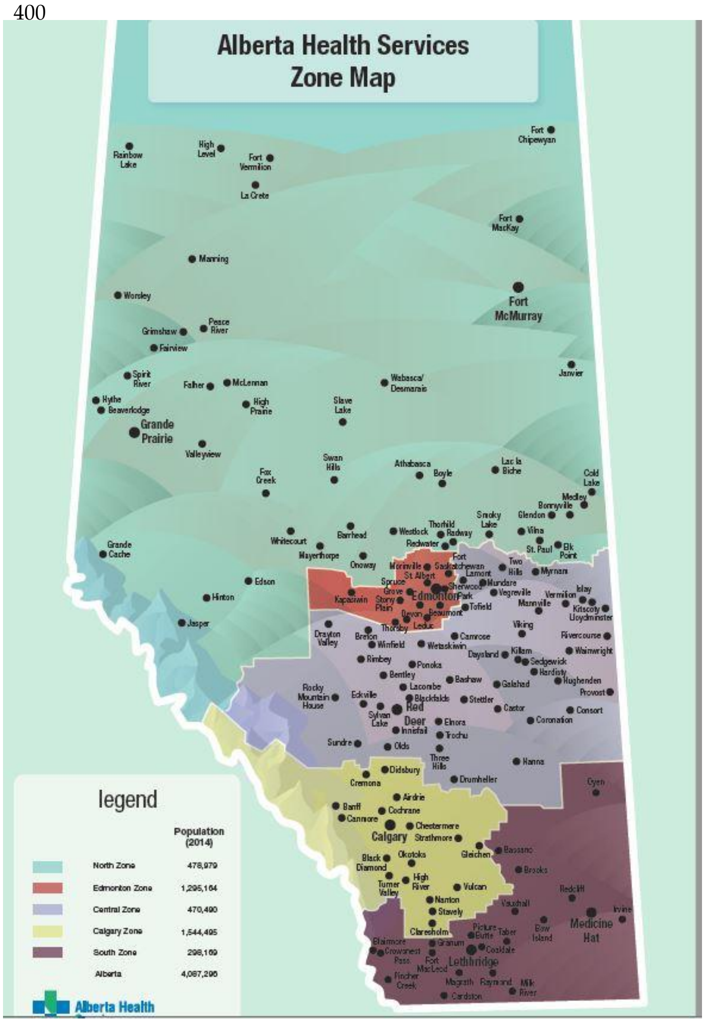

1. Which Alberta Health Zone do you live in? (See Map)
   ☐North Zone
   ☐Edmonton Zone
   ☐Central Zone
   ☐Calgary Zone
   ☐South Zone
   ☐Prefer not to answer/don't know.

2. How old are you?
   ☐Under 14
   ☐15 to 24
   ☐25 to 34
   ☐35 to 44
   ☐45 to 54
   ☐55 to 64
   ☐65 to 74
   ☐Over 75 years of age
   ☐Prefer not to answer/don't know

3. What is your gender? I describe myself as:
   ☐ man
   ☐ non-binary [insert text box], prefer not to say.
   ☐ woman
   ☐ prefer to self-describe as _______________________

Section 8 Your Turn!

1. Is there anything that you want to tell us about your caregiving role, dealing with the COVID-19 pandemic or what you need to continue caregiving in the COVID-19 pandemic and beyond.

____

____

____

May we contact you to participate in a focus group interview so that we can get an in-depth understanding of how COVID-19 has affected your caregiving role? If so please provide us with your name and email address, or phone number:

Name____

Email Address ____

Phone number ____

☐I would like to participate in a draw for two $50 VISA gift certificates. I have provided my contact information above.

☐I would like to participate in a draw for two $50 VISA gift certificates, but I DO Not want to participate in an interview. I have provided my contact information above.

Thank you so much for completing the survey. We really appreciate all the work that family caregivers do.

Please Return Survey to:

Dr. Jasneet Parmar /Dr. Sharon Anderson

6-40G University Terrace

8303 - 112 St NW

Edmonton AB T6G 2T4

sdanders@ualberta.ca

Supplementary Materials 2: Table Stages of thematic analysis

Step 1	Researchers familiarize themselves with data	Read transcripts and listen to the recordings. Make corrections in transcriptions, Make notes on tone, first impressions of the data.
Step 2	Identify preliminary codes	Identify interesting elements in data. Document impressions with notes.<br>Write memos on emerging themes.<br>Document connected elements.
Step 3	Find themes in the data	Look for and identify themes in the data.<br>Identify all data relevant to the themes.
Step 4	Finalize the themes	Finalize the themes. Check for data overlaps between the themes.
Step 5	Review each theme	Review the data to ensure that data fits each unique theme. Reread the transcriptions to ensure the overall data fits with the overall storyline and each theme.
Sept 6	Document and review documented analysis	Analyze the resultant documentation and the inferences drawn.

Supplementary Materials 3: CHERRIES checklist: The Checklist for Reporting Results of Internet E-Surveys

Design	Describe survey design	Section 2 Paragraph 1
IRB (Institutional Review Board) approval and informed consent process.	IRB approval.	Section 2 Paragraph 1 University Health Ethics Research Board.
	Informed consent.	Implied Consent Section 2 Paragraph 1 last sentence added.
	Data protection.	Section 2 Paragraph 1 REDCap secure data collection platform.
Development and pre-testing.	Development and testing.	Section 2 Paragraphe 1 Added. Questionnaire reviewed by research team and then not-for-profit partners. Online REDCap survey reviewed by 10 family caregivers.
	Open survey versus closed survey.	Added open survey Section 2 Paragraph 1 (highlighted).
	Contact mode.	Section 2 Paragraph 1.
Recruitment process and description of the sample having access to the questionnaire.	Advertising the survey.	Section 2 Paragraph 1.
	Web/Email.	Participants directed to REDCap link.
Survey administration.	Context.	Delivered on REDCAP, 4 people asked for paper copy, those responses entered manually.
	Mandatory/voluntary.	As per ethics, only first 2 qualifying questions were mandatory.
	Incentives.	Draw for 2, $50 gift cards, Explanation included in Ethics Information about the Survey (Supplementary File 1).
	Time/Date.	Section 2 Paragraph 1 June 21 to July 31, 2020.
	Randomization of items.	No.
	Adaptive questioning.	Yes, Branching questions in REDCap. e.g. questions about homecare or care location only to those answering yes to those situations.
	Number of Items.	Sections were displayed as one page in REDCap.
	Number of screens (pages).	8 sections/ 8 pages.
	Completeness check.	Yes, Completed by REDCap platform

	Review step.	Yes participants could review/change answers and REDCap download PDF of responses was enabled.
Response rates.	Unique site visitor.	As per ethics anonymity we did not gather IP addresses or use cookies.
	View rate (Ratio of unique survey visitors/unique site visitors).	Not possible to calculate.
	Participation rate (Ratio of unique visitors who agreed to participate/unique first survey page visitors),	Section 3 Results 1st paragraph 58.9% (highlighted),
	Completion rate (Ratio of users who finished the survey/users who agreed to participate),	Section 3 Results 1st paragraph 80.6% (Highlighted),
	Cookies used,	As per ethics anonymity we did not use cookies,
Preventing multiple entries from the same individual,	IP check,	As per ethics anonymity we did not use cookies,
	Log file analysis,	Checked for identical responses.
	Registration,	No,
Analysis,	Handling of incomplete questionnaires,	Those with less than ½ of the questions completed were excluded,
	Questionnaires submitted with an atypical timestamp,	None identified,
	Statistical correction,	No.
